# Identification of TAZ as the essential molecular switch in orchestrating SCLC phenotypic transition and metastasis

**DOI:** 10.1093/nsr/nwab232

**Published:** 2022-01-04

**Authors:** Yujuan Jin, Qiqi Zhao, Weikang Zhu, Yan Feng, Tian Xiao, Peng Zhang, Liyan Jiang, Yingyong Hou, Chenchen Guo, Hsinyi Huang, Yabin Chen, Xinyuan Tong, Jiayu Cao, Fei Li, Xueliang Zhu, Jun Qin, Dong Gao, Xin-Yuan Liu, Hua Zhang, Luonan Chen, Roman K Thomas, Kwok-Kin Wong, Lei Zhang, Yong Wang, Liang Hu, Hongbin Ji

**Affiliations:** State Key Laboratory of Cell Biology, Chinese Academy of Sciences, Shanghai 200031, China; Shanghai Institute of Biochemistry and Cell Biology, Chinese Academy of Sciences, Shanghai 200031, China; Center for Excellence in Molecular Cell Science, Chinese Academy of Sciences, Shanghai 200031, China; State Key Laboratory of Cell Biology, Chinese Academy of Sciences, Shanghai 200031, China; Shanghai Institute of Biochemistry and Cell Biology, Chinese Academy of Sciences, Shanghai 200031, China; Center for Excellence in Molecular Cell Science, Chinese Academy of Sciences, Shanghai 200031, China; Center for Excellence in Mathematical Sciences, National Center for Mathematics and Interdisciplinary Sciences, Key Laboratory of Management, Decision and Information System, Hua Loo-Keng Center for Mathematical Sciences, Academy of Mathematics and Systems Science, Chinese Academy of Sciences, Beijing 100190, China; State Key Laboratory of Cell Biology, Chinese Academy of Sciences, Shanghai 200031, China; Shanghai Institute of Biochemistry and Cell Biology, Chinese Academy of Sciences, Shanghai 200031, China; Center for Excellence in Molecular Cell Science, Chinese Academy of Sciences, Shanghai 200031, China; Shenzhen Key Laboratory of Translational Medicine of Tumor, Department of Cell Biology and Genetics, Shenzhen University Health Sciences Center, Shenzhen 518060, China; Shanghai Pulmonary Hospital, Tongji University, Shanghai 200092, China; Shanghai Chest Hospital, Shanghai Jiaotong University, Shanghai 200030, China; Zhongshan Hospital, Fudan University, Shanghai 200032, China; State Key Laboratory of Cell Biology, Chinese Academy of Sciences, Shanghai 200031, China; Shanghai Institute of Biochemistry and Cell Biology, Chinese Academy of Sciences, Shanghai 200031, China; Center for Excellence in Molecular Cell Science, Chinese Academy of Sciences, Shanghai 200031, China; State Key Laboratory of Cell Biology, Chinese Academy of Sciences, Shanghai 200031, China; Shanghai Institute of Biochemistry and Cell Biology, Chinese Academy of Sciences, Shanghai 200031, China; Center for Excellence in Molecular Cell Science, Chinese Academy of Sciences, Shanghai 200031, China; State Key Laboratory of Cell Biology, Chinese Academy of Sciences, Shanghai 200031, China; Shanghai Institute of Biochemistry and Cell Biology, Chinese Academy of Sciences, Shanghai 200031, China; Center for Excellence in Molecular Cell Science, Chinese Academy of Sciences, Shanghai 200031, China; State Key Laboratory of Cell Biology, Chinese Academy of Sciences, Shanghai 200031, China; Shanghai Institute of Biochemistry and Cell Biology, Chinese Academy of Sciences, Shanghai 200031, China; Center for Excellence in Molecular Cell Science, Chinese Academy of Sciences, Shanghai 200031, China; State Key Laboratory of Cell Biology, Chinese Academy of Sciences, Shanghai 200031, China; Shanghai Institute of Biochemistry and Cell Biology, Chinese Academy of Sciences, Shanghai 200031, China; Center for Excellence in Molecular Cell Science, Chinese Academy of Sciences, Shanghai 200031, China; Department of Pathology, School of Basic Medical Sciences, Fudan University, Shanghai 200032, China; State Key Laboratory of Cell Biology, Chinese Academy of Sciences, Shanghai 200031, China; Shanghai Institute of Biochemistry and Cell Biology, Chinese Academy of Sciences, Shanghai 200031, China; Center for Excellence in Molecular Cell Science, Chinese Academy of Sciences, Shanghai 200031, China; School of Life Science and Technology, Shanghai Tech University, Shanghai 200120, China; CAS Key Laboratory of Tissue Microenvironment and Tumor, CAS Center for Excellence in Molecular Cell Science, Shanghai Institute of Nutrition and Health Sciences, Chinese Academy of Sciences, Shanghai 200031, China; State Key Laboratory of Cell Biology, Chinese Academy of Sciences, Shanghai 200031, China; Shanghai Institute of Biochemistry and Cell Biology, Chinese Academy of Sciences, Shanghai 200031, China; Center for Excellence in Molecular Cell Science, Chinese Academy of Sciences, Shanghai 200031, China; State Key Laboratory of Cell Biology, Chinese Academy of Sciences, Shanghai 200031, China; Shanghai Institute of Biochemistry and Cell Biology, Chinese Academy of Sciences, Shanghai 200031, China; Center for Excellence in Molecular Cell Science, Chinese Academy of Sciences, Shanghai 200031, China; Laura and Isaac Perlmutter Cancer Center, New York University Langone Medical Center, New York, NY 10016, USA; State Key Laboratory of Cell Biology, Chinese Academy of Sciences, Shanghai 200031, China; Shanghai Institute of Biochemistry and Cell Biology, Chinese Academy of Sciences, Shanghai 200031, China; Center for Excellence in Molecular Cell Science, Chinese Academy of Sciences, Shanghai 200031, China; School of Life Science, Hangzhou Institute for Advanced Study, University of Chinese Academy of Sciences, Hangzhou 310024, China; Department of Translational Genomics, Center of Integrated Oncology Cologne-Bonn, Medical Faculty, University of Cologne, Cologne 50931, Germany; Department of Pathology, University Hospital Cologne, Cologne 50937, Germany; Laura and Isaac Perlmutter Cancer Center, New York University Langone Medical Center, New York, NY 10016, USA; State Key Laboratory of Cell Biology, Chinese Academy of Sciences, Shanghai 200031, China; Shanghai Institute of Biochemistry and Cell Biology, Chinese Academy of Sciences, Shanghai 200031, China; Center for Excellence in Molecular Cell Science, Chinese Academy of Sciences, Shanghai 200031, China; School of Life Science, Hangzhou Institute for Advanced Study, University of Chinese Academy of Sciences, Hangzhou 310024, China; Center for Excellence in Mathematical Sciences, National Center for Mathematics and Interdisciplinary Sciences, Key Laboratory of Management, Decision and Information System, Hua Loo-Keng Center for Mathematical Sciences, Academy of Mathematics and Systems Science, Chinese Academy of Sciences, Beijing 100190, China; School of Life Science, Hangzhou Institute for Advanced Study, University of Chinese Academy of Sciences, Hangzhou 310024, China; State Key Laboratory of Cell Biology, Chinese Academy of Sciences, Shanghai 200031, China; Shanghai Institute of Biochemistry and Cell Biology, Chinese Academy of Sciences, Shanghai 200031, China; Center for Excellence in Molecular Cell Science, Chinese Academy of Sciences, Shanghai 200031, China; State Key Laboratory of Cell Biology, Chinese Academy of Sciences, Shanghai 200031, China; Shanghai Institute of Biochemistry and Cell Biology, Chinese Academy of Sciences, Shanghai 200031, China; Center for Excellence in Molecular Cell Science, Chinese Academy of Sciences, Shanghai 200031, China; School of Life Science, Hangzhou Institute for Advanced Study, University of Chinese Academy of Sciences, Hangzhou 310024, China

**Keywords:** small cell lung cancer, SWI/SNF complex, TAZ, phenotypic transition, metastasis

## Abstract

Small-cell lung cancer (SCLC) is a recalcitrant cancer characterized by high metastasis. However, the exact cell type contributing to metastasis remains elusive. Using a *Rb1*^L/L^*/Trp53*^L/L^ mouse model, we identify the NCAM^hi^CD44^lo/–^ subpopulation as the SCLC metastasizing cell (SMC), which is progressively transitioned from the non-metastasizing NCAM^lo^CD44^hi^ cell (non-SMC). Integrative chromatin accessibility and gene expression profiling studies reveal the important role of the SWI/SNF complex, and knockout of its central component, *Brg1*, significantly inhibits such phenotypic transition and metastasis. Mechanistically, TAZ is silenced by the SWI/SNF complex during SCLC malignant progression, and its knockdown promotes SMC transition and metastasis. Importantly, ectopic TAZ expression reversely drives SMC-to-non-SMC transition and alleviates metastasis. Single-cell RNA-sequencing analyses identify SMC as the dominant subpopulation in human SCLC metastasis, and immunostaining data show a positive correlation between TAZ and patient prognosis. These data uncover high SCLC plasticity and identify TAZ as the key molecular switch in orchestrating SCLC phenotypic transition and metastasis.

## INTRODUCTION

Small-cell lung cancer (SCLC) is characterized by very poor prognosis, with ∼15% global lung cancer incidence and a five-year survival lower than 7% [1]. This can be largely attributed to the highly metastatic capability of SCLC. Most SCLC patients are initially diagnosed at extensive stage, characterized by nearby lung and/or distant metastases. Therefore, exploration of the mechanisms involved in SCLC metastasis is urgently needed so as to provide helpful insights into clinical management.

Previous studies have shown that ∼90% of human SCLC harbors concurrent inactivating mutations or deletions of *Rb1* and *Trp53* [2]. Homozygous deletion of these two alleles in mouse lung epithelia promotes SCLC development and dramatic metastasis, which closely recapitulates human SCLC in the clinic [3]. Mouse SCLC in the *Rb1*^L/L^/*Trp53*^L/L^ (*RP*) model typically expresses neuroendocrine markers including neuronal cell adhesion molecule (NCAM) and achaete-scute complex homolog 1 (ASCL1), and frequently metastasizes into distant organs [3]. Concurrent deletion of *P130*, an *Rb*-related gene, or *Pten* in the RP model, significantly accelerates malignant progression and SCLC metastasis [4,5]. Moreover, upregulated Nuclear Factor I B (NFIB) expression is found to promote SCLC metastasis through increasing the accessibility of global chromatin [6–8].

SCLCs are characterized by high heterogeneity [9–15]. It is proposed that human SCLC is composed of four different subtypes based on lineage-related transcription factors including ASCL1, NEUROD1, YAP and POU2F3 [14]. More recently, an inflamed SCLC subtype has been identified with a good response to immunotherapy [16]. Similar heterogeneity has also been found in mouse SCLC, e.g. the CD24^hi^CD44^lo^EpCAM^hi^ subpopulation from the *RP* model is identified as harboring a strong capability to form tumors in allograft assay [11]. Moreover, mouse SCLC is found to contain the neuroendocrine (NE) and non-neuroendocrine (non-NE) subpopulations according to distinct growth patterns in culture, with the NE subtype growing as suspension and the non-NE as adhesion [9]. The NE cells frequently express neuroendocrine markers including NCAM, synaptophysin (SYP) and ASCL1. In contrast, the non-NE cells tend to express mesenchymal markers such as VIMENTIN and CD44 [9]. It has been reported that the synergetic cooperation between NE and non-NE subpopulations is necessary for SCLC metastasis whereas neither subtype could metastasize on its own [9]. Therefore, the exact population responsible for SCLC metastasis still remains unknown.

The switch/sucrose-non-fermentable (mSWI/SNF) complexes, including canonical BRG1/BRM-associated factor (BAF), polybromo-associated BAF (PBAF) and non-canonical BAF (ncBAF), are essential for chromatin remodeling [17,18]. All three complexes contain a core ATPase subunit, e.g. BRG1 (Brahma/SWI2-related gene 1, also called SMARCA4), which catalyzes the hydrolysis of ATP [18]. Previous studies reveal that the SWI/SNF complexes tend to function as tumor suppressors during cancer development. Consistently, a high incidence of BRG1 inactivating mutation is detected in multiple cancer types including lung cancer [19]. Previous studies show that BRG1 promotes cell cycle arrest and senescence through the retinoblastoma pathway in cancer cells [20,21]. Interestingly, recent studies have also indicated an oncogenic role of BRG1. For example, BRG1 promotes pancreatic intraepithelial neoplasia (PanIN) development and gastric cancer metastasis [22–24]. In SCLC, BRG1 is preferentially required for cancer progression when MAX (Myc-associated factor) is inactivated [25]. These findings indicate that BRG1 might function as tumor suppressor or oncogenic driver in a cell-type- or genetic-context-dependent manner.

The Hippo pathway is initially defined as an important pathway during organ size control, and functions mainly via the synergetic interaction between the transcription factor TEAD1-4 and transcriptional co-activator YAP/TAZ (WWTR1) [26]. The oncogenic activities of YAP/TAZ have been well documented in multiple epithelial cancers [26–31]. It is well known that YAP/TAZ sustains self-renewal and tumor-initiating capability, and promotes cancer malignant progression and metastasis through epithelial-to-mesenchymal transition (EMT) [30]. The latest studies also reveal that YAP/TAZ might function as a tumor suppressor [32–34]. For instance, YAP expression is downregulated in breast cancer and knockdown of YAP promotes cancer cell migration and invasiveness [35]. Moreover, we have previously found that YAP acts as the barrier for adenocarcinoma-to-squamous-carcinoma transdifferentiation (AST) as well as for lung squamous-cell carcinoma progression [33,36]. Nonetheless, the exact role of YAP/TAZ during SCLC metastasis has not been characterized yet.

We here identify NCAM^hi^CD44^lo/–^ cells as the major subpopulation responsible for SCLC metastasis. Moreover, this subpopulation is progressively transitioned from the non-metastatic NCAM^lo^CD44^hi^ cells via the SWI/SNF-complex-mediated TAZ silencing. Our data highlight the important link between epigenetically regulated TAZ and SCLC plasticity and metastasis.

## RESULTS

### Identification of the NCAM^hi^CD44^lo/–^ subpopulation as SCLC metastasizing cells

**Figure 1.**
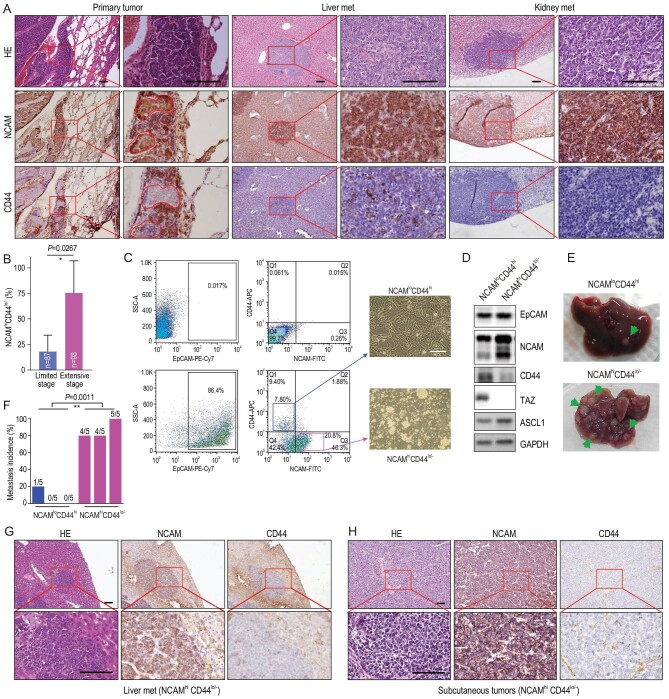
Identification of the NCAM^hi^CD44^lo/–^ cells as SCLC metastasizing cells in the *RP* mouse model. (A) Representative photos of Hematoxylin-Eosin (HE) staining, NCAM and CD44 IHC staining of primary tumors, liver and kidney metastases (met) from the RP mouse model. The intra-tumor heterogeneity of primary tumors is shown in high magnification. The marked areas in high magnification panels indicate the NCAM^hi^CD44^lo/–^ expression pattern. Scale bars, 100 μm. (B) Statistic analyses of the NCAM^hi^CD44^lo/–^ tumors at limited stage (no overt distant organ metastasis) and extensive stage (overt metastasis) in the RP model. The NCAM^hi^CD44^lo/–^ tumors were defined when the lesions contained >50% of cells showing NCAM^hi^ and CD44^lo/–^ expression. Limited stage: 87 tumors from 4 mice were analyzed; extensive stage: 98 tumors from 4 mice were analyzed. Data are shown as mean ± S.E.M. *P* value was calculated by unpaired two-tailed *t* test. (C) Flow cytometry (FACS) analyses of primary tumors from the RP mouse model using antibodies towards EpCAM, NCAM and CD44. The tumor cells without primary antibody incubation are shown as negative control (top panels). The NCAM^lo^CD44^hi^ and NCAM^hi^CD44^lo/–^ cells were sorted and cultured *in vitro* and the representative cell growth photos are shown on the right. Scale bar, 100 μm. (D) Western blot detection of EpCAM, NCAM, CD44, ASCL1 and TAZ expression in established NCAM^lo^CD44^hi^ and NCAM^hi^CD44^lo/–^ SCLC primary cell lines. (E and F) Representative photos (E) and the incidence (F) of liver metastasis in nude mice subcutaneously transplanted with primary NCAM^lo^CD44^hi^ or NCAM^hi^CD44^lo/–^ cells derived from the RP mouse model. Data are shown from three independent experiments (*n* = 5 mice for each experiment). The ratio of mice with liver metastasis was also indicated. *P* value was calculated by unpaired two-tailed *t* test. (G and H) Representative photos of HE staining, NCAM and CD44 IHC staining of (G) liver metastases, and (H) subcutaneous tumors in nude mice transplanted with NCAM^hi^CD44^lo/–^ cell lines. Scale bars, 100 μm.

To study SCLC heterogeneity during cancer malignant progression and metastasis, we first performed immunohistochemistry (IHC) staining in *RP* tumors using NE marker NCAM and mesenchymal marker CD44. In primary *RP* tumors, we indeed observed the heterogeneous expression pattern of these two markers intra-tumorally and inter-tumorally (Fig. 1A and Table S1). We found that the percentage of NCAM^hi^CD44^lo/–^ tumors, defined with over 50% of cancer cells highly expressing NCAM and with low or no CD44 expression [37], increased with malignant progression and metastasis (Fig. 1B, Fig. S1A and Table S1). Consistently, we found that distant organ metastases such as liver and kidney metastases uniformly exhibited the NCAM^hi^CD44^lo/–^ expression pattern (Fig. 1A). These data indicate that the NCAM^hi^CD44^lo/–^ subpopulation might be responsible for SCLC metastasis.

To test this, we then used Fluorescence Activated Cell Sorting (FACS) to isolate the NCAM^hi^CD44^lo/–^ and NCAM^lo^CD44^hi^ subpopulations from primary *RP* tumors (Fig. 1C). Genotyping analyses confirmed the concurrent deletion of *Rb1* and *Trp53* in both subpopulations (Fig. S1B). We found that the NCAM^hi^CD44^lo/–^ cells grew in culture as oncospheres with a suspension growth pattern (Fig. 1C), similar to classical human SCLC cell lines. In contrast, the NCAM^lo^CD44^hi^ cells grew as adhesion (Fig. 1C). Moreover, a higher ASCL1 level was detected in the NCAM^hi^CD44^lo/–^ subpopulation (Fig. 1D). We then subcutaneously transplanted 5 × 10^6^ cells from either the NCAM^hi^CD44^lo/–^ or NCAM^lo^CD44^hi^ subpopulation into nude mice and waited for up to 10 weeks for distant organ metastasis analyses. Both subpopulations formed subcutaneous tumors at 100% in allograft assay, with comparable tumor growth (Fig. S1C and D). In contrast, the metastasis analyses revealed a huge difference. Most mice (13 out of 15) from the NCAM^hi^CD44^lo/–^ group had spontaneous metastases in the liver whereas only 1 out of 15 mice from the NCAM^lo^CD44^hi^ group displayed distant metastasis (Fig. 1E and F). The liver metastases from the NCAM^hi^CD44^lo/–^ group exhibited a characteristic marker expression pattern, similar to subcutaneous tumors (Fig. 1G and H). These data demonstrate that the NCAM^hi^CD44^lo/–^ cells are mainly responsible for SCLC metastasis. We hereafter refer to the NCAM^hi^CD44^lo/–^ and NCAM^lo^CD44^hi^ subpopulations as SCLC metastasizing cell (SMC) and non-SCLC metastasizing cell (non-SMC), respectively.

### Phenotypic transition from non-SMC to SMC contributes to SCLC metastasis

**Figure 2.**
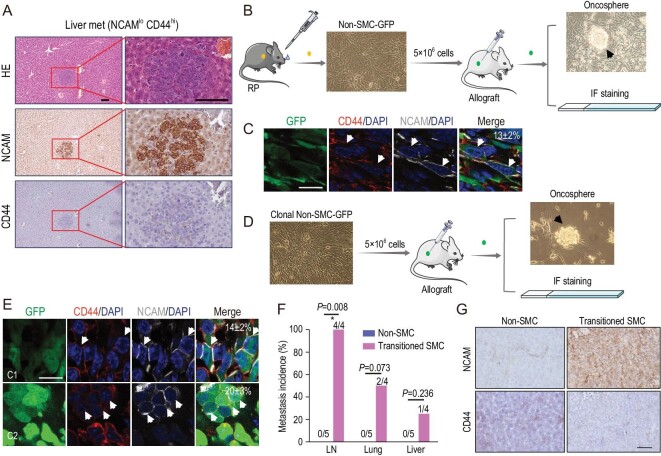
Phenotypic transition from non-SMC to SMC contributes to SCLC metastasis. (A) Representative photos for HE staining, NCAM and CD44 IHC staining in the only liver metastasis from nude mice subcutaneously transplanted with the NCAM^lo^CD44^hi^ cell lines (non-SMC) derived from the RP mouse model. Scale bars, 100 μm. (B) Experimental scheme to test phenotypic transition from non-SMC to SMC. Primary non-SMC was derived from the RP mouse model and ectopically expressed GFP, and then used for subcutaneous transplantation in nude mice. The subcutaneous tumors were analyzed through oncosphere formation, and NCAM and CD44 IF staining. Oncospheres in cell culture were indicated. (C) Representative photos of NCAM and CD44 IF staining in subcutaneous tumors from nude mice transplanted with non-SMC-GFP cells. The NCAM^hi^CD44^lo/–^ subpopulation indicated by white arrows were microscopically counted and the mean ratio of NCAM^hi^CD44^lo/–^ cells is indicated in the top right corner. Scale bar, 25 μm. Data are shown as mean ± S.E.M. (D) Experimental scheme to test the potential phenotypic transition using single-cell-derived clonal non-SMC-GFP. The subcutaneous tumors were then analyzed through oncosphere formation and NCAM and CD44 IF staining. Oncospheres in cell culture were indicated. (E) Representative photos of NCAM and CD44 IF staining in clonal non-SMC-GFP subcutaneous tumors. C1: clone #1; C2: clone #2. The NCAM^hi^CD44^lo/–^ subpopulation indicated by white arrows was microscopically counted and the ratio of NCAM^hi^CD44^lo/–^ cells is indicated in the top right corner. Scale bar, 25 μm. Data are shown as mean ± S.E.M. (F) Statistical analyses of the incidence of lymph node (LN), lung and liver metastases in nude mice subcutaneously transplanted with transitioned SMC or non-SMC, which were derived from the clonal non-SMC-GFP subcutaneous tumors. *n* = 4 mice for transitioned SMC group and *n* = 5 mice for paired non-SMC group. *P* values were calculated by Pearson chi-square test. (G) Representative photos of NCAM and CD44 IHC staining of mouse livers in (F). The livers from paired non-SMC showed no metastasis. Scale bar, 100 μm.

Consistent with SMC metastatic tumors, the liver metastasis lesion from the non-SMC allograft assay also exhibited the NCAM^hi^CD44^lo/–^ expression pattern (Fig. 2A and Fig. S2). We speculated that there might exist phenotypic transition from non-SMC to SMC during SCLC malignant progression. To test this, we established a non-SMC cell line stably expressing GFP, termed non-SMC-GFP, and performed a subcutaneous allograft assay (Fig. 2B). Immunofluorescence (IF) staining in allograft tumors revealed that ∼13 ± 2% GFP-positive cancer cells displayed the NCAM^hi^CD44^lo/–^ pattern whereas the rest remained as a non-SMC expression pattern (Fig. 2C and Table S2). Consistently, both suspension and adhesion growth patterns were observed when these allograft tumors were cultured *in vitro* (Fig. 2B). To further confirm such transition, we picked single-cell clones from non-SMC-GFP cells and performed allograft assay with the clonal non-SMC-GFP cell lines (Fig. 2D). Similarly, we found that these subcutaneous tumors also displayed the NCAM^hi^CD44^lo/–^ pattern, ranging from 14 ± 2% to 20 ± 3% (Fig. 2E and Table S2). A mixed growth pattern was also observed in culture (Fig. 2D). We further isolated the transitioned SMC with NCAM^hi^CD44^lo/–^ pattern and tested its metastasis capability using allograft assay. In contrast to no overt metastases in the non-SMC group, multiple distant organ metastases, e.g. lymph node, lung and liver metastases, were detectable in the transitioned SMC group (Fig. 2F and G). We found that the liver metastases also displayed the NCAM^hi^CD44^lo/–^ pattern (Fig. 2G). These data together convincingly proved the transition from non-SMC to SMC and highlighted the important role of such phenotypic transition in SCLC metastasis.

### Brg1 knockout inhibits SMC phenotypic transition and SCLC metastasis

**Figure 3.**
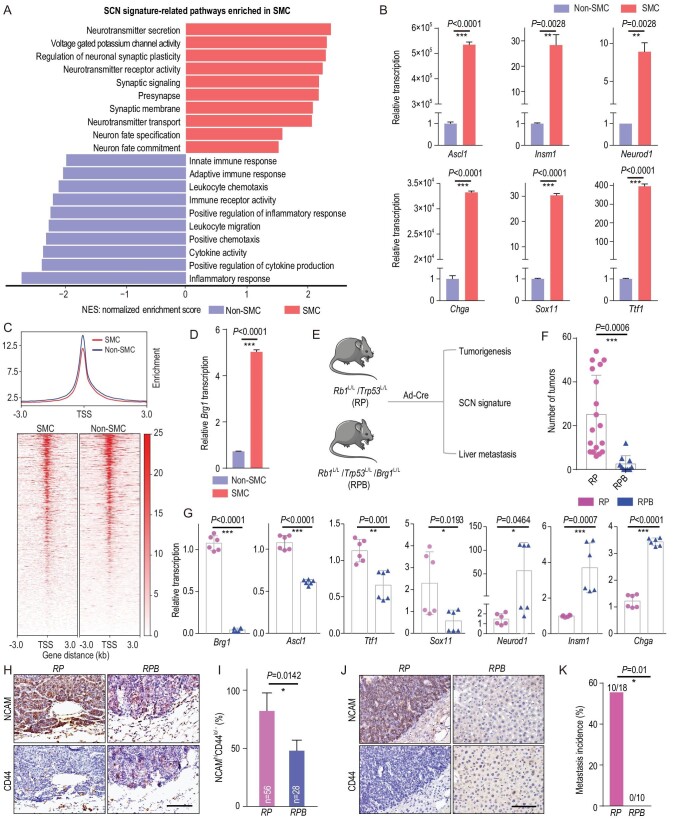
Knockout of *Brg1* in the *RP* mouse model significantly abrogates SMC phenotypic transition and SCLC metastasis. (A) The enrichment of small-cell neuroendocrine (SCN) signature-related pathways in SMC and the enrichment of immune-related pathways in non-SMC. NES, normalized enrichment score. (B) Real-time PCR detection of SCN-signature-related genes including *Ascl1*, *Insm1*, *Neurod1*, *Chga*, *Sox11* and *Ttf1* in SMC vs. non-SMC. Data are shown as mean ± S.E.M. *P* values were calculated by unpaired two-tailed *t* test. (C) Trend plot (top) and heat map (bottom) showing ATAC-seq signal over 6 kb regions centered at the transcription start sites (TSS) in SMC and non-SMC. (D) Real-time PCR detection of *Brg1* expression in SMC vs. non-SMC. (E) Schematic illustration of the comparative analyses of *Rb1*^L/L^/*Trp53*^L/L^ (*RP*) and *Rb1*^L/L^/*Trp53*^L/L^/*Brg1*^L/L^ (*RPB*) mice. (F) Statistical analyses of primary tumor numbers in *RP* and *RPB* mice at 32 weeks after Ad-Cre treatment. *n* = 18 mice for the *RP* group, *n* = 10 mice for the *RPB* group. Data are shown as mean ± S.E.M. *P* value was calculated by unpaired two-tailed *t* test. (G) Real-time PCR detection of *Brg1* and the SCN-signature-related genes in primary tumors from *RP* and *RPB* mice. *n* = 2 mice for each group. Data are shown as mean ± S.E.M. *P* values were calculated by unpaired two-tailed *t* test. (H) Representative photos of NCAM and CD44 IHC staining in primary tumors from *RP* and *RPB* mice at 32 weeks after Ad-Cre treatment. Scale bar, 100 μm. (I) Statistical analyses of the percentage of primary tumors with an NCAM^hi^CD44^lo/–^ expression pattern in *RP* and *RPB* mice. The NCAM^hi^CD44^lo/–^ tumors were defined when the lesions contained >50% of cells showing NCAM^hi^ and CD44^lo/–^ expression. A total of 56 tumors from 3 *RP* mice and 28 tumors from 4 *RPB* mice were analyzed. Data are shown as mean ± S.E.M. *P* value was calculated by unpaired two-tailed *t* test. (J) Representative photos of NCAM and CD44 IHC staining in livers of *RP* and *RPB* mice. The livers from *RPB* mice contained no metastasis. Scale bar, 100 μm. (K) Liver metastasis incidence in *RP* and *RPB* mice at 32 weeks after Ad-Cre treatment. *n* = 18 mice for the *RP* group, *n* = 10 mice for the *RPB* group. *P* value was calculated by Pearson chi-square test.

To further explore the molecular mechanisms underlying non-SMC-to-SMC transition, we performed RNA sequencing and comparatively analyzed the gene expression profiling of SMC and non-SMC. The small-cell neuroendocrine (SCN) signature has been recently established as an important index for SCLC metastasis [38]. Interestingly, we found a significant enrichment of SCN-signature-related pathways in SMC whereas non-SCN-related pathways (immune-related pathways) were enriched in non-SMC (Fig. 3A and Tables S3 and S4). Real-time PCR data further confirmed the increased expression of SCN signature genes, including *Ascl1*, *Insm1*, *Neurod1*, *Chga*, *Sox11* and *Ttf1*, in SMC (Fig. 3B). These data might partially explain the high metastasis capability of SMC.

Epigenetic alterations have been implicated in cancer plasticity [39,40]. We performed the assay for transposase-accessible chromatin with next-generation sequencing (ATAC-seq) to determine the global chromatin accessibility of SMC and non-SMC. Our analyses on transcription start sites also revealed an overall reduced signal in the active promoter regions of SMC (Fig. 3C). The total reads of ATAC-seq for SMC and non-SMC were ∼36 million and 27 million, respectively. Chromatin remodelers, such as SWI/SNF complex, are critical for regulating chromatin architecture and accessibility [18]. We found that multiple members of the SWI/SNF complex, including the central catalytic ATPase *Brg1*, were markedly dysregulated between these two subpopulations (Fig. S3A). Real-time PCR quantification further confirmed the significant upregulation of *Brg1* in SMC (Fig. 3D).

To test whether *Brg1* is involved in the phenotypic transition and SCLC metastasis, we generated the *Rb1*^L/L^/*Trp53*^L/L^/*Brg1*^L/L^ (*RPB*) mouse cohort and performed comparative analyses of tumorigenesis, SCN signature enrichment and metastasis in parallel with the *RP* model (Fig. 3E). We found that *Brg1* knockout significantly reduced the tumor number (Fig. 3F, and Fig. S3B and C). Moreover, several SCN-signature-related genes, including *Ascl1*, *Ttf1* and *Sox11*, were significantly downregulated in *RPB* tumors (Fig. 3G and Fig. S3B). IHC staining of the NCAM and CD44 showed that the percentage of primary tumors with an SMC expression pattern was also decreased in the *RPB* group (Fig. 3H and I and Table S5). Notably, no liver metastasis was detected in the *RPB* group in contrast to ∼50% incidence in the *RP* model (Fig. 3J and K). These data support the conclusion that the SWI/SNF complex is important for SMC phenotypic transition and SCLC metastasis.

### Epigenetic silencing of TAZ by SWI/SNF complex in SMC

**Figure 4.**
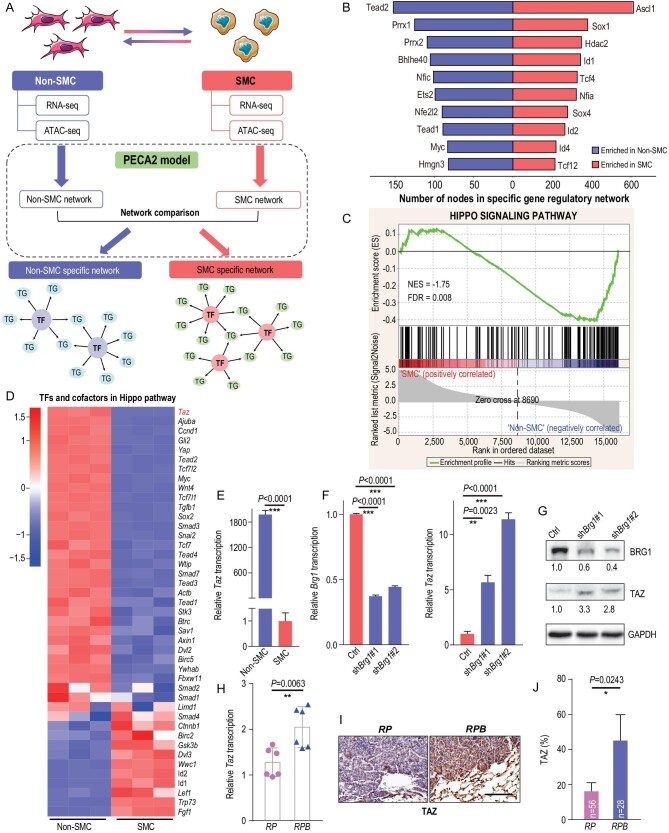
TAZ is epigenetically silenced by the SWI/SNF complex in SMC. (A) Schematic illustration of the integrative analyses of RNA-seq and ATAC-seq in SMC and non-SMC. Specific TF networks in SMC and non-SMC were constructed according to the PECA2 model (see details in Materials and Methods). TG, target genes. (B) Enriched TFs in SMC and non-SMC through integrative analyses of ATAC-seq and RNA-seq data ranked according to the numbers of dysregulated target genes. (C) Gene set enrichment analysis (GSEA) plot of the Hippo signaling pathway in SMC vs. non-SMC. (D) Heat map of RNA-seq data showing the relative expression of TFs and cofactors in the Hippo pathway in SMC vs. non-SMC. (E) Real-time PCR detection of Taz in SMC and non-SMC. Data are shown as mean ± S.E.M. *P* value was calculated by unpaired two-tailed *t* test. (F) Real-time PCR detection of *Brg1* and *Taz* in SMC with or without *Brg1* knockdown. *Gapdh* served as the internal control. Data are shown as mean ± S.E.M. *P* values were calculated by unpaired two-tailed *t* test. (G) Western blot detection of BRG1 and TAZ levels in SMC with or without *Brg1* knockdown. GAPDH served as the internal control. (H) Real-time PCR detection of *Taz* in primary tumors from *RP* and *RPB* mice at 32 weeks after Ad-Cre treatment. *Gapdh* served as the internal control. *n* = 2 for each group. Data are shown as mean ± S.E.M. *P* value was calculated by unpaired two-tailed *t* test. (I) Representative photos of TAZ IHC staining in primary tumors from *RP* and *RPB* mice at 32 weeks after Ad-Cre treatment. Scale bar, 100 μm. (J) Percentage of TAZ positive tumors in *RP* vs. *RPB* mice at 32 weeks after Ad-Cre treatment; 56 tumors from 3 *RP* mice and 28 tumors from 4 *RPB* mice were analyzed. Data are shown as mean ± S.E.M. *P* value was calculated by unpaired two-tailed *t* test.

To identify the downstream mediator of the SWI/SNF complex in contribution to SCLC phenotypic transition and metastasis, we first constructed the dysregulated transcriptional factor (TF) network through the integrative analyses of RNA-seq and ATAC-seq data as previously described [41]. We found that *Ascl1* and *Tead2* were top-ranked TFs with the highest number of dysregulated target genes in SMC and non-SMC respectively (Fig. 4A and B, Figs S4 and S5, and Table S6). ASCL1 is known as the pioneering TF that initializes neuronal reprogramming and is also included in the SCN biomarker genes [42]. TEAD family members are important TFs that function with cofactor YAP/TAZ in cancer malignant progression [30,43,44]. Gene set enrichment analysis revealed that the Hippo pathway was significantly enriched in non-SMC (Fig. 4C). Moreover, *Taz/Yap* stood out as top hits among the dysregulated components of the Hippo pathway (Fig. 4D). Using real-time PCR, we further confirmed the decreased expression of *Taz/Yap* in SMC vs. non-SMC cells (Fig. 4E and Fig. S3D).

We further asked whether *Taz/Yap* expression was regulated by *Brg1*. We found that *Brg1* knockdown in SMC cells resulted in a significant upregulation of TAZ expression whereas the expression of YAP was downregulated (Fig. 4F and G and Fig. S3E). Such upregulation of TAZ was also detectable in *RPB* tumors in comparison to *RP* tumors (Fig. 4H and J and Table S5). Moreover, we observed an obvious decreased chromatin accessibility at the promoter region of *Taz* in SMC (Fig. S3F), which might explain the reduced TAZ expression (Fig. 1D). A similar but lesser degree of chromatin accessibility change was also observed at the *Yap* promoter region in SMC (Fig. S3F). In support of this, TAZ level was obviously downregulated in primary tumors at extensive stage (Fig. S1A). Moreover, knockdown of *Arid1a* or *Arid2*, another two important components of the SWI/SNF complex, obviously upregulated TAZ expression in SMC cells (Fig. S3G and H). However, YAP expression was only slightly upregulated with *Arid2* knockdown, or even downregulated after *Arid1a* knockdown in SMC (Fig. S3G and H). Moreover, we performed BRG1 Chromatin Immunoprecipitation real-time quantitative PCR (ChIP-qPCR) analysis and found that BRG1 could bind to the promoter region of *Taz* (Fig. S3I). These results together demonstrate that TAZ is silenced during non-SMC-to-SMC transition through SWI/SNF-complex-mediated epigenetic reprogramming.

### TAZ knockdown promotes non-SMC-to-SMC transition and accelerates SCLC metastasis

**Figure 5.**
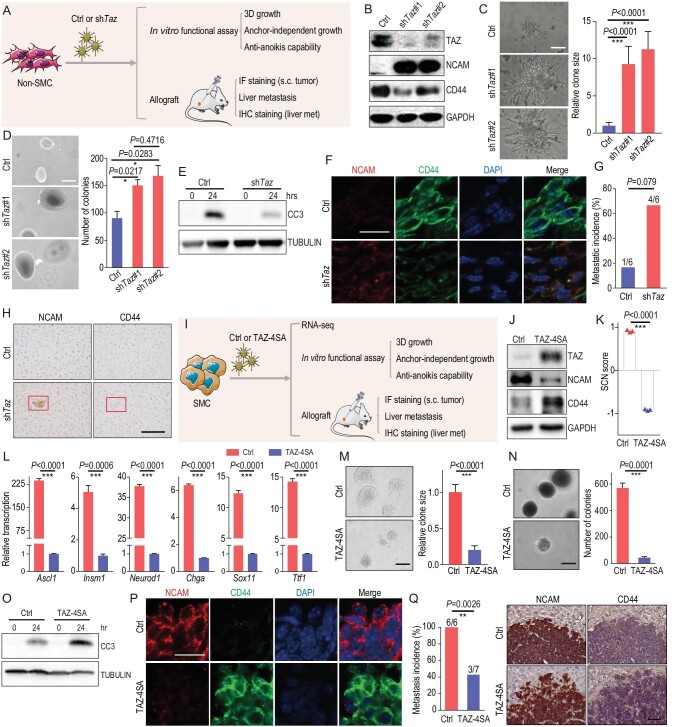
TAZ functions as a critical molecular switch in regulating the phenotypic transition and SCLC metastasis. (A) Schematic illustration of the comparative analyses of non-SMC with or without *Taz* knockdown. (B) Western blot detection of TAZ, NCAM and CD44 levels in non-SMC with or without *Taz* knockdown. GAPDH served as the internal control. (C) Representative photos of the matrigel invasiveness of non-SMC with or without *Taz* knockdown (left). The statistical analyses of the clone sizes were performed using Image J software. Scale bars, 100 μm. *P* values were calculated by unpaired two-tailed *t* test. (D) Representative photos (left) and number (right) of the soft-agar colonies of non-SMC with or without *Taz* knockdown. Scale bars, 100 μm. Data are shown as mean ± S.E.M. *P* value was calculated by unpaired two-tailed *t* test. (E) Western blot detection of cleaved caspase3 (CC3) in anti-anoikis assay of non-SMC with or without *Taz* knockdown. TUBULIN served as the internal control. (F) Representative photos of NCAM and CD44 IF staining in subcutaneous tumors from nude mice transplanted with non-SMC with or without *Taz* knockdown. Scale bar, 25 μm. (G) Metastasis incidence and (H) representative photos of NCAM and CD44 IHC staining in livers from nude mice transplanted by non-SMC with or without *Taz* knockdown. *n* = 6 for each group. *P* value was calculated by Pearson chi-square test. Scale bar, 100 μm. (I) Schematic illustration of the comparative analyses of SMC with or without ectopic TAZ-4SA expression. (J) Western blot detection of TAZ, NCAM and CD44 levels in SMC with or without ectopic TAZ-4SA expression. GAPDH served as the internal control. (K) SCN score of SMC with or without ectopic TAZ-4SA expression. Data are shown as mean ± S.E.M. *P* value was calculated by unpaired two-tailed *t* test. (L) Real-time PCR detection of the SCN-signature-related genes in SMC with or without ectopic TAZ-4SA expression. *Gapdh* served as the internal control. Data are shown as mean ± S.E.M. *P* values were calculated by unpaired two-tailed *t* test. (M) Representative photos of the matrigel invasiveness of SMC with or without ectopic TAZ-4SA expression (left). The statistical analyses of the clone sizes were performed using Image J software. Scale bar, 100 μm. *P* values were calculated by unpaired two-tailed *t* test. (N) Representative photos (left) and statistical analyses (right) of soft-agar colonies of SMC with or without ectopic TAZ-4SA expression. Scale bar, 100 μm. Data are shown as mean ± S.E.M. *P* value was calculated by unpaired two-tailed *t* test. (O) Western blot detection of CC3 in an anti-anoikis assay of SMC with or without ectopic TAZ-4SA expression. TUBULIN served as the internal control. (P) Representative photos of NCAM and CD44 IF staining in subcutaneous tumors from nude mice transplanted with SMC with or without ectopic TAZ-4SA expression. Scale bar, 25 μm. (Q) Metastasis incidence (left) and representative photos of NCAM and CD44 IHC staining of liver metastasis (right) in nude mice transplanted with SMC with or without ectopic TAZ-4SA expression. *n* = 6 mice for the control group, *n* = 7 mice for the TAZ-4SA group. Scale bar, 100 μm. *P* value was calculated by Pearson chi-square test.

To explore the function of TAZ in phenotype transition and SCLC metastasis, we performed *Taz* knockdown in non-SMC for allograft assay (Fig. 5A). We found that *Taz* knockdown in non-SMC upregulated NCAM and SCN-related genes, whereas it downregulated CD44, without a dramatic effect upon *Yap* expression (Fig. 5B and Fig. S6A–C). Moreover, *Taz* knockdown also promoted the invasiveness in matrigel, colony formation in soft agar and anti-anoikis capability of non-SMC (Fig. 5C and E). IF staining of allograft tumors showed that *Taz* knockdown promoted the appearance of the NCAM^hi^CD44^lo/–^ pattern, resembling the SMC-derived tumors (Fig. 5F and Fig. S6D). Importantly, knockdown of *Taz* in non-SMC promoted distant organ metastasis (Fig. 5G). IHC staining further confirmed that these metastases displayed the SMC expression pattern (Fig. 5H). These data together demonstrate that TAZ downregulation promotes phenotypic transition from non-SMC to SMC and SCLC metastasis.

### Ectopic TAZ expression reversely promotes the transition from SMC to non-SMC and alleviates SCLC metastasis

To test if the phenotypic transition from non-SMC to SMC is reversible, we ectopically expressed a constitutive activated TAZ mutant (TAZ-4SA) [30] in SMC (Fig. 5I). We found that the downstream targets of TAZ, including *Cyr61*, *Ctgf*, *Areg*, *Vim* and *Axl*, were significantly upregulated after ectopic TAZ-4SA expression in SMC (Fig. S6E). Ectopic TAZ-4SA but not YAP-5SA [33] expression in SMC dramatically downregulated NCAM and upregulated CD44 expression *in vitro*, indicative of the potential reversible transition from SMC to non-SMC (Fig. 5J, Fig. S6F, and Tables S7 and S8). Moreover, the SCN score and related gene expression also decreased after ectopic TAZ-4SA expression (Fig. 5K and L and Tables S7 and S8). Functional assays showed that TAZ-4SA expression markedly suppressed the matrigel invasiveness, colony formation in soft agar and anti-anoikis capability of SMC (Fig. 5M and O). IF staining also showed that ectopic TAZ-4SA expression promoted the non-SMC expression pattern in comparison to SMC-derived subcutaneous tumors (Fig. 5P and Fig. S6G). More importantly, ectopic TAZ-4SA expression significantly suppressed the liver metastases of SMC (Fig. 5Q). Furthermore, all the liver metastases consistently showed no TAZ expression (Fig. S6H). These findings support the conclusion that ectopic *TAZ* expression promotes reverse transition from SMC to non-SMC and alleviates SCLC metastasis.

### Low TAZ level is associated with SCN signature enrichment and predicts poor prognosis of SCLC patients

**Figure 6.**
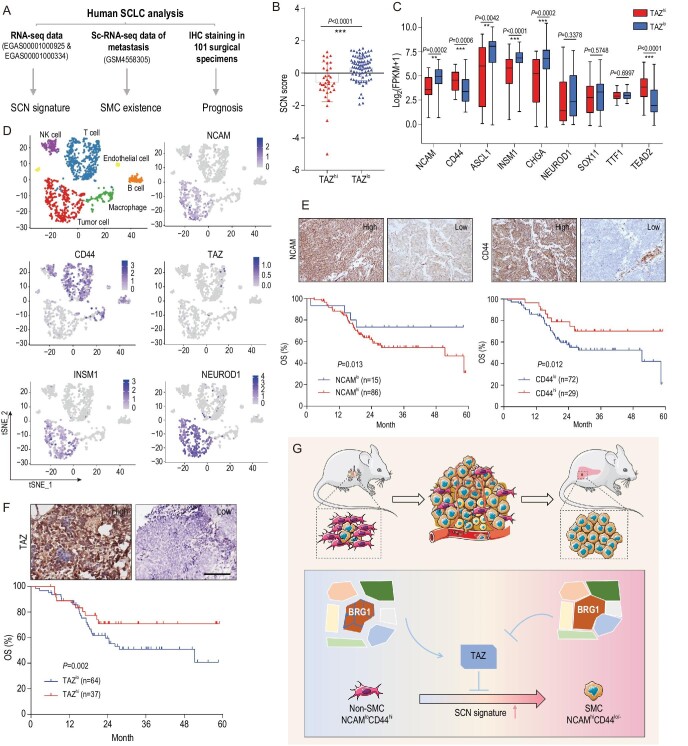
Low TAZ level is correlated with SCN signature enrichment and predicts poor prognosis of SCLC patients. (A) Schematic illustration of the analyses of human SCLC specimens. (B) SCN score of human SCLC specimens with high or low TAZ mRNA level. The RNA-seq data were downloaded from a public database (GSE69091 and EGAS00001000334). Data are shown as mean ± S.E.M. *P* value was calculated by unpaired two-tailed *t* test. (C) Correlation between individual SCN-signature-related genes, CD44 or TEAD2 expression with high or low TAZ level in human SCLC (GSE69091 and EGAS00001000334). Data are shown as mean ± S.E.M. *P* values were calculated by unpaired two-tailed *t* test. (D) Clustering and the NCAM, CD44, TAZ, NEUROD1 and INSM1 expression of the single cell sequencing data (GSM4558305) of a liver biopsy from an SCLC patient. (E and F) Representative photos of (E) NCAM, CD44 and (F) TAZ IHC staining in Chinese SCLC specimens (top) and survival curves of high or low expression of NCAM, CD44 or TAZ with overall survival (OS) (bottom). Scale bar, 100 μm. *P* values were calculated by Kaplan-Meier analysis with log-rank test. (G) Working model illustrating the essential role of SWI/SNF-complex-mediated TAZ expression in controlling the phenotypic transition from non-SMC to SMC and SCLC metastasis. TAZ, which is epigenetically silenced by the SWI/SNF complex, functions as a critical molecular switch during the phenotypic transition from non-SMC to SMC and SCLC metastasis. Disruption of the SWI/SNF complex through BRG1 knockout promotes TAZ upregulation and thus inhibits the phenotypic transition and cancer metastasis.

To evaluate whether our findings are clinically relevant, we downloaded a public RNA-sequencing dataset of 112 human SCLCs [2,45] and analyzed the correlation between *TAZ* and SCN signature, and single-cell sequencing data of liver metastasis [15], to detect whether SMC exists in metastatic lesion, and collected 101 Chinese surgical specimens for prognosis analyses (Fig. 6A). Bioinformatic analyses showed that human SCLC with low *TAZ* expression (*TAZ*^lo^) displays a significantly higher SCN score (Fig. 6B and Table S9), indicative of strong metastasis capability. The SCN-signature-related pathways, including positive regulation of neurotransmitter transport, neurotransmitter secretion and synaptic vesicle membrane, were significantly enriched in *TAZ*^lo^ SCLC (Fig. S7A). Consistently, most SCN-signature-related genes, including *ASCL1*, *INSM1* and *CHGA*, were significantly increased in *TAZ*^lo^ SCLC samples (Fig. 6C). Moreover, *NCAM* was increased, and *CD44* was decreased in *TAZ*^lo^ SCLC specimens (Fig. 6C), indicative of the SMC pattern of these samples. *TEAD* also decreased in these *TAZ*^lo^ samples (Fig. 6C). Also, we observed a negative correlation between the SCN-signature-related genes and *TAZ*, and a positive correlation between *CD44*, *TEAD2* and *TAZ* (Fig. S7B and Table S9).

We further took advantage of the Ireland *et al*. single-cell RNA-sequencing data derived from SCLC liver metastasis [15]. Interestingly, we found that most SCLC metastatic cells showed high expression of *NCAM* with concurrent low expression of *CD44*, resembling the SMC pattern (Fig. 6D). Moreover, these cells showed high expression of the SCN signature markers *INSM1* and *NEUROD1*, similar to the SMC in the RP model (Fig. 6D). Importantly, low or no *TAZ* expression was detected in these metastasis cells (Fig. 6D), confirming the silence of *TAZ* in metastasis.

Lastly, we put together a patient cohort containing 101 Chinese SCLC surgical specimens for immunostaining analyses of NCAM, CD44 and TAZ. Most of these patients were at limited stage without distant metastases. We found that high NCAM or low CD44 levels were significantly associated with worse patient overall survival (OS) (Fig. 6E and Table S10). Moreover, TAZ^lo^ patients also showed a worse overall survival (Fig. 6F). These data together provide strong clinical evidence in support of our findings of SMC in the *RP* model.

## DISCUSSION

SCLC is the most lethal form of lung cancer, characterized by highly metastatic capacity. A growing body of evidence based on mouse models has demonstrated that SCLC is highly heterogeneous with distinct subpopulations playing different roles during malignant progression and metastasis [5–15]. In this study, we identify the NCAM^hi^CD44^lo/–^ cells in the *RP* model as the SCLC metastasizing cells. We further reveal that the SMCs are progressively transitioned from non-SMCs during SCLC malignant progression and metastasis. Our data further show that the SWI/SNF-complex-mediated epigenetic downregulation of TAZ is essential for driving such a phenotype transition. Moreover, TAZ activation is sufficient to drive the reverse transition from SMC to non-SMC and thus alleviate SCLC metastasis. With the support of clinical specimen analyses, our data demonstrate that the NCAM^hi^CD44^lo/–^ cells are mainly responsible for SCLC metastasis and the SWI/SNF-TAZ axis importantly orchestrates SCLC plasticity and metastasis (Fig. 6G).

To assess SCLC heterogeneity in the *RP* model, we use both NE marker NCAM and mesenchymal marker CD44 to do the immunostaining and FACS analyses, and identify the NCAM^hi^CD44^lo/–^ cells as the SCLC metastasizing cells. A previous study shows that mouse SCLC cells contain both NE and non-NE subpopulations [9]. However, neither subpopulation alone can metastasize and a synergetic cooperation is necessary for distant organ metastasis [9]. In contrast, our data show that the NCAM^hi^CD44^lo/–^ cells harbor strong metastasis capability in allograft assay, and the tumors metastasize into multiple distant organs including the lymph node, lung and liver. Since the SMC defined here also expresses classical NE biomarkers, we reason that the NCAM^hi^CD44^lo/–^ cells might belong to the NE subpopulation, but with higher metastasis potential. In other words, the NCAM^hi^CD44^lo/–^ cells might represent the highly metastatic subpopulation of the NE subtype. In addition, we used primary NCAM^hi^CD44^lo/-^ cells and demonstrated their robust metastatic capability, whereas Calbo *et al*. [9] employed tumor-derived cell lines for metastasis assays. Future efforts looking into the heterogeneity of the NE subtype will hopefully uncover more subpopulations linked to SCLC malignant progression and metastasis.

We also find that phenotypic transition from non-SMC to SMC contributes to SCLC metastasis, which closely links cancer plasticity and malignant progression. Indeed, recent data also show, during SCLC drug resistance acquisition, that Notch signaling promotes the transition from an NE to non-NE subtype and thus provides a niche for resisting drug treatment [10]. A similar transition from NE to non-NE subtypes has also been found in another recent study [15]. Metastasis and drug resistance are two major hurdles in clinical SCLC management. Understanding molecular mechanisms involved in the phenotypic transition in these two important events will hopefully provide a solid base for the development of a novel therapeutic strategy to treat SCLC in the clinic.

Through integrative analyses of gene expression profiling and chromatin accessibility, we find that SWI/SNF complexes play an important role during non-SMC-to-SMC transition. Although the SWI/SNF complex is generally considered to be tumor suppressive [46], our results indicate that this complex has a different role in SCLC progression. Knockout of its ATPase BRG1 inhibits such phenotypic transition and cancer metastasis, indicating the oncogenic function of the SWI/SNF complex as well as BRG1 in SCLC. In agreement with our observation, a previous study reported that BRG1 is important for the activation of NE transcriptional programs to upregulate MYC targets, and depletion of BRG1 strongly hinders cell growth, specifically in MAX-deficient SCLC tumors [25]. Consistently, we find that BRG1 knockdown suppresses neuronal gene expression and several SCN-signature-related genes in the SMC subpopulation. Likewise, the dual roles of ARID1A have also been revealed in cancer [47]. Thus, the exact function of the SWI/SNF complex and its subunit as tumor suppressor or oncogenic driver might be cell-type or genetic-context dependent and vary with the type of malignancy.

We further find that TAZ is an important downstream mediator of the SWI/SNF complex during SCLC phenotypic transition. Although both YAP and TAZ are significantly upregulated in non-SMC, only TAZ is significantly upregulated when *Brg1* is knocked down in SMC. Similar findings are also observed when *Arid1a* or *Arid2* is knocked down. Consistently, *Brg1* knockout in an *RP* mouse upregulates TAZ and significantly inhibits SMC appearance and SCLC metastasis. Moreover, we find that low *TAZ* expression is associated with SCN signature enrichment. In agreement with these observations, previous studies have shown that high YAP/TAZ expression correlates with decreased NE markers [48], and YAP loss defines NE differentiation [49]. Meanwhile, NE lineage markers are dominant in the SCN signature, which is significantly associated with SCLC malignant progression and metastasis [38,50–52]. Of course, considering the redundant function and concurrent decrease of YAP and TAZ, it remains possible that YAP may also contribute to SCLC phenotypic transition and metastasis albeit independent of the SWI/SNF complex. Future efforts will be necessary to clarify the detailed regulatory mechanisms underlying YAP expression during SCLC phenotypic transition and metastasis.

Our findings from loss-of-function and gain-of-function experiments support the tumor-suppressive role of TAZ in SCLC. YAP/TAZ is well established as oncogenic driver. Nonetheless, accumulated evidence has recently revealed the tumor-suppressive function of YAP/TAZ in multiple cancer types [53]. For instance, YAP restricts Wnt signals during intestinal regeneration, which results in rapid loss of intestinal crypts, and YAP loss promotes hyperplasia and microadenoma development [54]. In hematological cancer, low YAP level prevents nuclear ABL1-induced apoptosis and rescued YAP expression triggers cell death [55]. Another study shows that the growth inhibitory effect caused by LATS1/2 deletion is due to uncontrolled activation of YAP in colon cancer [56]. A recent report demonstrates that LATS1/2 promotes breast cancer cell growth through inhibition of YAP/TAZ [34]. Our findings with regard to the tumor-suppressive function of TAZ are also supported by clinical specimen analyses. Single-cell RNA-sequencing data support that the cancer cells from SCLC liver metastasis mainly display the SMC expression pattern and these metastatic cells show low or no expression of TAZ. Moreover, low TAZ level is significantly associated with poor patient survival. These data together support that TAZ works as a tumor suppressor in controlling SCLC plasticity and metastasis.

## MATERIALS AND METHODS

### RP and RPB mouse cohort generation, maintenance and analyses

Mice were housed in a specific pathogen-free environment at the Shanghai Institute of Biochemistry and Cell Biology, and treated in accordance with protocols conforming to the ARRIVE guidelines and approved by the Institutional Animal Care and Use Committee of the Shanghai Institutes for Biological Sciences, Chinese Academy of Sciences (approval number: IBCB0011). Conditional knockout mice including *Trp53*^L/L^, *Rb1*^L/L^ [3] and *Brg1*^L/L^ [57] alleles were generously provided by Drs. Tyler Jacks, Ronald A. DePinho and Pierre Chambon. Mice were crossed to obtain *Rb1*^L/L^/*Trp53*^L/L^ (*RP*) and *Rb1*^L/L^/*Trp53*^L/L^/*Brg1*^L/L^ (*RPB*) cohorts. All experimental mice were maintained on a mixed genetic background as previously described [58]. Mice at 6–8 weeks old were treated with Adenovirus-CMV-Cre recombinase (Ad-Cre, 2 × 10^6^ p.f.u.) by intratracheal intubation [59] to allow for Cre-lox mediated recombination of floxed alleles. Mouse tumors were used for immunostaining, FACS analyses, genomic DNA extraction and genotyping as previously described [3,57]. The primer sequences are shown in the supplementary data.

### Statistical analysis

Statistical analyses were carried out using SPSS 16.0 or GraphPad Prism 5/7 software (San Diego, CA). The significance of differences was determined using a two-tailed Student's *t* test or chi-square test. Kaplan-Meier analysis with log-rank test was used to assess patients’ survival between subgroups. *P* value <0.05 was considered to be statistically significant.

## DATA AVAILABILITY

Sequence data have been deposited in Gene Expression Omnibus (GEO) with the primary accession codes GSE158091 (ATAC-seq of SMC and non-SMC), GSE158290 (RNA-seq of SMC and non-SMC) and GSE158293 (RNA-seq of SMC-Ctrl and SMC-TAZ-4SA).

## Supplementary Material

nwab232_Supplemental_Files
