# Correction to: Identification of TAZ as the essential molecular switch in orchestrating SCLC phenotypic transition and metastasis

**DOI:** 10.1093/nsr/nwac224

**Published:** 2023-12-28

**Authors:** Yujuan Jin, Qiqi Zhao, Weikang Zhu, Yan Feng, Tian Xiao, Peng Zhang, Liyan Jiang, Yingyong Hou, Chenchen Guo, Hsinyi Huang, Yabin Chen, Xinyuan Tong, Jiayu Cao, Fei Li, Xueliang Zhu, Jun Qin, Dong Gao, Xin-Yuan Liu, Hua Zhang, Luonan Chen, Roman K Thomas, Kwok-Kin Wong, Lei Zhang, Yong Wang, Liang Hu, Hongbin Ji

**Affiliations:** State Key Laboratory of Cell Biology, Chinese Academy of Sciences, Shanghai 200031, China; Shanghai Institute of Biochemistry and Cell Biology, Chinese Academy of Sciences, Shanghai 200031, China; Center for Excellence in Molecular Cell Science, Chinese Academy of Sciences, Shanghai 200031, China; State Key Laboratory of Cell Biology, Chinese Academy of Sciences, Shanghai 200031, China; Shanghai Institute of Biochemistry and Cell Biology, Chinese Academy of Sciences, Shanghai 200031, China; Center for Excellence in Molecular Cell Science, Chinese Academy of Sciences, Shanghai 200031, China; School of Life Science and Technology, Shanghai Tech University, Shanghai 200120, China; Center for Excellence in Mathematical Sciences, National Center for Mathematics and Interdisciplinary Sciences, Key Laboratory of Management, Decision and Information System, Hua Loo-Keng Center for Mathematical Sciences, Academy of Mathematics and Systems Science, Chinese Academy of Sciences, Beijing 100190, China; State Key Laboratory of Cell Biology, Chinese Academy of Sciences, Shanghai 200031, China; Shanghai Institute of Biochemistry and Cell Biology, Chinese Academy of Sciences, Shanghai 200031, China; Center for Excellence in Molecular Cell Science, Chinese Academy of Sciences, Shanghai 200031, China; Shenzhen Key Laboratory of Translational Medicine of Tumor, Department of Cell Biology and Genetics, Shenzhen University Health Sciences Center, Shenzhen 518060, China; Shanghai Pulmonary Hospital, Tongji University, Shanghai 200092, China; Shanghai Chest Hospital, Shanghai Jiaotong University, Shanghai 200030, China; Zhongshan Hospital, Fudan University, Shanghai 200032, China; State Key Laboratory of Cell Biology, Chinese Academy of Sciences, Shanghai 200031, China; Shanghai Institute of Biochemistry and Cell Biology, Chinese Academy of Sciences, Shanghai 200031, China; Center for Excellence in Molecular Cell Science, Chinese Academy of Sciences, Shanghai 200031, China; State Key Laboratory of Cell Biology, Chinese Academy of Sciences, Shanghai 200031, China; Shanghai Institute of Biochemistry and Cell Biology, Chinese Academy of Sciences, Shanghai 200031, China; Center for Excellence in Molecular Cell Science, Chinese Academy of Sciences, Shanghai 200031, China; State Key Laboratory of Cell Biology, Chinese Academy of Sciences, Shanghai 200031, China; Shanghai Institute of Biochemistry and Cell Biology, Chinese Academy of Sciences, Shanghai 200031, China; Center for Excellence in Molecular Cell Science, Chinese Academy of Sciences, Shanghai 200031, China; State Key Laboratory of Cell Biology, Chinese Academy of Sciences, Shanghai 200031, China; Shanghai Institute of Biochemistry and Cell Biology, Chinese Academy of Sciences, Shanghai 200031, China; Center for Excellence in Molecular Cell Science, Chinese Academy of Sciences, Shanghai 200031, China; State Key Laboratory of Cell Biology, Chinese Academy of Sciences, Shanghai 200031, China; Shanghai Institute of Biochemistry and Cell Biology, Chinese Academy of Sciences, Shanghai 200031, China; Center for Excellence in Molecular Cell Science, Chinese Academy of Sciences, Shanghai 200031, China; Department of Pathology, School of Basic Medical Sciences, Fudan University, Shanghai 200032, China; State Key Laboratory of Cell Biology, Chinese Academy of Sciences, Shanghai 200031, China; Shanghai Institute of Biochemistry and Cell Biology, Chinese Academy of Sciences, Shanghai 200031, China; Center for Excellence in Molecular Cell Science, Chinese Academy of Sciences, Shanghai 200031, China; School of Life Science and Technology, Shanghai Tech University, Shanghai 200120, China; CAS Key Laboratory of Tissue Microenvironment and Tumor, CAS Center for Excellence in Molecular Cell Science, Shanghai Institute of Nutrition and Health Sciences, Chinese Academy of Sciences, Shanghai 200031, China; State Key Laboratory of Cell Biology, Chinese Academy of Sciences, Shanghai 200031, China; Shanghai Institute of Biochemistry and Cell Biology, Chinese Academy of Sciences, Shanghai 200031, China; Center for Excellence in Molecular Cell Science, Chinese Academy of Sciences, Shanghai 200031, China; State Key Laboratory of Cell Biology, Chinese Academy of Sciences, Shanghai 200031, China; Shanghai Institute of Biochemistry and Cell Biology, Chinese Academy of Sciences, Shanghai 200031, China; Center for Excellence in Molecular Cell Science, Chinese Academy of Sciences, Shanghai 200031, China; Laura and Isaac Perlmutter Cancer Center, New York University Langone Medical Center, New York, NY 10016, USA; State Key Laboratory of Cell Biology, Chinese Academy of Sciences, Shanghai 200031, China; Shanghai Institute of Biochemistry and Cell Biology, Chinese Academy of Sciences, Shanghai 200031, China; Center for Excellence in Molecular Cell Science, Chinese Academy of Sciences, Shanghai 200031, China; School of Life Science, Hangzhou Institute for Advanced Study, University of Chinese Academy of Sciences, Hangzhou 310024, China; Department of Translational Genomics, Center of Integrated Oncology Cologne-Bonn, Medical Faculty, University of Cologne, Cologne 50931, Germany; Department of Pathology, University Hospital Cologne, Cologne 50937, Germany; Laura and Isaac Perlmutter Cancer Center, New York University Langone Medical Center, New York, NY 10016, USA; State Key Laboratory of Cell Biology, Chinese Academy of Sciences, Shanghai 200031, China; Shanghai Institute of Biochemistry and Cell Biology, Chinese Academy of Sciences, Shanghai 200031, China; Center for Excellence in Molecular Cell Science, Chinese Academy of Sciences, Shanghai 200031, China; School of Life Science, Hangzhou Institute for Advanced Study, University of Chinese Academy of Sciences, Hangzhou 310024, China; Center for Excellence in Mathematical Sciences, National Center for Mathematics and Interdisciplinary Sciences, Key Laboratory of Management, Decision and Information System, Hua Loo-Keng Center for Mathematical Sciences, Academy of Mathematics and Systems Science, Chinese Academy of Sciences, Beijing 100190, China; School of Life Science, Hangzhou Institute for Advanced Study, University of Chinese Academy of Sciences, Hangzhou 310024, China; State Key Laboratory of Cell Biology, Chinese Academy of Sciences, Shanghai 200031, China; Shanghai Institute of Biochemistry and Cell Biology, Chinese Academy of Sciences, Shanghai 200031, China; Center for Excellence in Molecular Cell Science, Chinese Academy of Sciences, Shanghai 200031, China; State Key Laboratory of Cell Biology, Chinese Academy of Sciences, Shanghai 200031, China; Shanghai Institute of Biochemistry and Cell Biology, Chinese Academy of Sciences, Shanghai 200031, China; Center for Excellence in Molecular Cell Science, Chinese Academy of Sciences, Shanghai 200031, China; School of Life Science and Technology, Shanghai Tech University, Shanghai 200120, China; School of Life Science, Hangzhou Institute for Advanced Study, University of Chinese Academy of Sciences, Hangzhou 310024, China

This is a correction to: Yujuan Jin, Qiqi Zhao, Weikang Zhu, Yan Feng, Tian Xiao, Peng Zhang, Liyan Jiang, Yingyong Hou, Chenchen Guo, Hsinyi Huang, Yabin Chen, Xinyuan Tong, Jiayu Cao, Fei Li, Xueliang Zhu, Jun Qin, Dong Gao, Xin-Yuan Liu, Hua Zhang, Luonan Chen, Roman K Thomas, Kwok-Kin Wong, Lei Zhang, Yong Wang, Liang Hu, Hongbin Ji, Identification of TAZ as the essential molecular switch in orchestrating SCLC phenotypic transition and metastasis, *National Science Review*, Volume 9, Issue 7, July 2022, nwab232, https://doi.org/10.1093/nsr/nwab232

In the originally published version of this manuscript, affiliation ‘School of Life Science and Technology, ShanghaiTech University, Shanghai 200120, China’ was inadvertently omitted from authors Hongbin Ji and Qiqi Zhao. This error has now been corrected.

Originally listed as affiliation 10, ‘School of Life Science and Technology, ShanghaiTech University, Shanghai 200120, China’, has now been been listed as affiliation 4, and affiliations 5, 6, 7, 8 and 9 have been renumbered accordingly. The published manuscript has now been corrected.

